# Physical inactivity induces insulin resistance in plantaris muscle through protein tyrosine phosphatase 1B activation in mice

**DOI:** 10.3389/fphys.2023.1198390

**Published:** 2023-06-14

**Authors:** Saori Kakehi, Yoshifumi Tamura, Shin-ichi Ikeda, Naoko Kaga, Hikari Taka, Yuya Nishida, Ryuzo Kawamori, Hirotaka Watada

**Affiliations:** ^1^ Department of Metabolism and Endocrinology, Tokyo, Japan; ^2^ Sportology Center, Tokyo, Japan; ^3^ Division of Proteomics and Biomolecular Science, Juntendo University Graduate School of Medicine, Tokyo, Japan

**Keywords:** insulin resistance, physical inactivity, high fat diet, skeletal muscle, PTP1B

## Abstract

Inactivity causes insulin resistance in skeletal muscle and exacerbates various lifestyle-related diseases. We previously found that 24-h hindlimb cast immobilization (HCI) of the predominantly slow-twitch soleus muscle increased intramyocellular diacylglycerol (IMDG) and insulin resistance by activation of lipin1, and HCI after a high-fat diet (HFD) further aggravated insulin resistance. Here, we investigated the effects of HCI on the fast-twitch–predominant plantaris muscle. HCI reduced the insulin sensitivity of plantaris muscle by approximately 30%, and HCI following HFD dramatically reduced insulin sensitivity by approximately 70% without significant changes in the amount of IMDG. Insulin-stimulated phosphorylation levels of insulin receptor (IR), IR substrate-1, and Akt were reduced in parallel with the decrease in insulin sensitivity. Furthermore, tyrosine phosphatase 1B (PTP1B), a protein known to inhibit insulin action by dephosphorylating IR, was activated, and PTP1B inhibition canceled HCI-induced insulin resistance. In conclusion, HCI causes insulin resistance in the fast-twitch–predominant plantaris muscle as well as in the slow-twitch–predominant soleus muscle, and HFD potentiates these effects in both muscle types. However, the mechanism differed between soleus and plantaris muscles, since insulin resistance was mediated by the PTP1B inhibition at IR in plantaris muscle.

## Introduction

Skeletal muscle accounts for approximately 40% of the human body mass and 90% of whole-body glucose uptake during hyper-insulinemic euglycemic clamp (DeFronzo et al., 1985). Thus, skeletal muscle is considered to be the most important tissue with regard to insulin-induced glucose disposal and hence the maintenance of glucose homeostasis. Indeed, insulin resistance in skeletal muscle has been identified as a major cause of metabolic syndrome and type 2 diabetes (DeFronzo, 2004; Petersen et al., 2012).

Insulin resistance in skeletal muscle is known to be caused by a high-fat diet (HFD) and physical inactivity (Fung et al., 2000; Dunstan et al., 2005; Ford et al., 2005; Healy et al., 2007; Harris et al., 2009). While exercise increases insulin sensitivity through various mechanisms (Hayashi et al., 1997; Rattigan et al., 2001; Rose and Richter, 2005; Geiger et al., 2006; Frosig et al., 2007; Lira et al., 2010), little is known about how physical inactivity reduces insulin sensitivity. In this regard, we have focused on the possibility that the accumulation of intramyocellular lipids in skeletal muscle causes insulin resistance. We recently found that 24 h of physical inactivity caused intracellular diacylglycerol (DG) accumulation in soleus muscle, which is composed mainly of slow-twitch muscle fibers, through activation of the lipid metabolism enzyme lipin1. In this situation, DG-induced PKCε activation and insulin resistance were observed, and HFD potentiated these effects (Kakehi et al., 2021).

Skeletal muscle is a heterogeneous tissue comprised of slow- and fast-twitch muscle fibers, each of which accounts for approximately 50% of muscle fibers in humans (Sato et al., 1984; Lexell et al., 1988; Sjostrom et al., 1991; Pette and Staron, 2000). Compared to slow-twitch fibers, fast-twitch fibers are characterized by slower rates of protein synthesis and degradation, as well as less atrophy caused by physical inactivity (Macpherson et al., 2011; von Walden et al., 2012; Sandona et al., 2012). In addition, the degree of intracellular lipid accumulation in skeletal muscle caused by HFD varies according to the type of muscle fiber. Prolonged (16- to 24-week) HFD resulted in significantly greater intramuscular lipid accumulation in fast-twitch than in slow-twitch muscle (Han et al., 1997; Masgrau et al., 2012; Messa et al., 2020; Umek et al., 2021). These findings suggest that the effects of physical inactivity on lipid accumulation and insulin sensitivity in skeletal muscle may differ between muscles composed of different fiber types. However, it is still unclear whether physical inactivity causes intracellular DG accumulation and insulin resistance in skeletal muscles other than the soleus.

Based on this background, we investigated the effect of short-term (24-h) physical inactivity in fast-twitch–predominant plantaris muscle (Goodman et al., 2012). As a model of short-term physical inactivity, we applied 24-h hindlimb cast immobilization (HCI) to mice that received a normal-fat diet (NFD) or a HFD and evaluated intramyocellular lipids and insulin signaling pathways in plantaris muscle.

## Methods

### Animals and experimental design

The present study complied with the principles and guidelines of the Japanese Council on Animal Care, and it was approved by the Committee for Animal Research of Juntendo University. C57BL/6J male mice (8–9 weeks old) were obtained from Charles River Laboratory (Portage, MI, United States) and were acclimatized for 1 week in an air-conditioned room with a 12:12-h light–dark cycle. The mice were divided into two groups, consisting of the NFD control group (12% fat) (CRF-1, Oriental yeast CO., LTD., Tokyo, Japan) and the HFD group (60% fat: soybean oil, 5%; lard oil, 55%) (product number D12494, Research Diet, Tokyo, Japan). Both groups were fed *ad libitum* for 2 weeks. On the last day of the dietary intervention, one hindlimb of each mouse was immobilized with a cast for 24 h. While the mice were anesthetized with 2% isoflurane (Merck Millipore, Darmstadt, Germany) inhalation, the HCI procedure was performed using a plaster bandage to keep the ankle joint in a fully extended position. To avoid any systemic effect of HCI on muscle metabolism, the opposite hindlimb was not casted and was used as a control after each dietary condition. Twenty-4 hours after immobilization, mice were administered anesthesia using 2% isoflurane and both plantar muscles were carefully removed. Mice were euthanized by cervical dislocation.

### Materials and reagents

Anti-insulin receptor substrate 1 (IRS1) antibody (06-248), phosphotyrosine (4G10) antibody (16-316), and anti–phospho Ser307-IRS1 antibody (05-1087) were obtained from Merck Millipore (Darmstadt, Germany). Anti-AKT antibody (9272), anti–phospho Ser473-AKT antibody (9271), anti–phospho Ser636/639-IRS-1 antibody (2388), anti–phospho Ser1101-IRS-1 antibody (2385), and anti–insulin receptor (IR) β antibody (3025) were obtained from Cell Signaling Technology (Danvers, MA, United States). Anti–protein tyrosine phosphatase 1B (PTP1B) antibody (ab252928) was obtained from Abcam (Cambridge, United Kingdom), and 3-(3,5-dibromo-4-hydroxybenzoyl)-2-ethyl-N-[4-[(2-thiazolylamino) sulfonyl] phenyl]-6-benzofuransulfonamide, a PTP1B inhibitor, was obtained from Santa Cruz Biotechnology (Dallas, TX, United States). Human recombinant insulin (Humulin R) was purchased from Eli Lilly (Indianapolis, IN, United States).

### *Ex vivo* muscle incubation and 2-deoxyglucose (2-DOG) uptake

*Ex vivo* incubation and 2-DOG uptake measurements were performed as described previously (Ikeda et al., 2013). Briefly, dissected plantaris muscles were pre-incubated for 30 min at 37°C in 3 mL Krebs-Riger-Bicarbonate (KRB) buffer containing 8 mM D-glucose. At the end of the pre-incubation period, the muscles were transferred to fresh KRB buffer containing 8 mM D-glucose with or without 450 μU/mL insulin and incubated for 20 min at 37°C. Following the incubation period, the muscles were rinsed in 3 mL KRB buffer containing 8 mM D-mannitol for 10 min at 30°C, and then transport was measured in 2 mL KRB buffer containing 1 mM 2-deoxy-D-[1,2-^3^H]-glucose (1.5 μCi/mL) and 7 mM D-[^14^C]-mannitol (0.3 μCi/mL) for 10 min at 30°C. All buffers were continuously gassed with 95% O_2_/5% CO_2_. To terminate transport, muscles were dipped in ice-cold KRB buffer. Muscles were blotted on filter paper, trimmed, and processed by incubation in 420 μL 1 N NaOH for 5 min at 100°C. Neutralization was performed with 70 μL 6 N HCl, and particulates were precipitated by centrifugation. The radioactivity of aliquots of digested muscle protein was determined by liquid scintillation counting for dual labels, and 2-DOG uptake was calculated as previously described (Higaki et al., 2008). For Western blotting, muscle was incubated *ex vivo* in the same manner as mentioned above except without radioisotopes. For PTP1B inhibition in *ex vivo* muscle incubation, 10 μM of PTP1B cell-permeable inhibitor (Hughes et al., 2015; Zhu et al., 2021) was preincubated with dissected plantaris muscle for 30 min at 37°C, followed by insulin stimulation. After all incubation periods were complete, muscles were frozen in liquid nitrogen and stored at −80°C until assay.

### Immunoprecipitation

Plantaris muscle was homogenized in RIPA buffer (50 mM Tris, 150 mM NaCl, 1 mM EDTA, 0.5% sodium deoxycholate, 1% Nonidet P-40, 1 μM aprotinin, 10 μM leupeptin, 0.1 μM phenylmethylsulfonyl fluoride, 20 mM sodium fluoride, 20 mM glycerolphosphate, and 1 mM sodium orthovanadate (pH 7.4)). The lysates were centrifuged at 800 *g* for 20 min at 4°C to remove insoluble material. The supernatants were incubated with the indicated antibodies, after which the immune complexes were precipitated with Dynabeads Protein G (Thermo Fisher Scientific, Waltham, MA, United States). The immunoprecipitates were subjected to SDS-PAGE and analyzed by Western blot analysis.

### Western blotting

For Western blotting, muscle samples were incubated *ex vivo* in the same manner as mentioned above except for the addition of the radioisotopes. Then, protein was extracted from the samples using lysis buffer (50 mM Tris-HCl, 150 mM NaCl, 0.1% Triton X-100, 1 mM EDTA, 1 mM NaF, pH 8.0) with Halt Protease and Phosphatase Inhibitor Cocktail (Thermo Fisher Scientific) and 0.1 mM PMSF. Ten micrograms of total proteins were separated by sodium dodecyl sulfate-polyacrylamide gel electrophoresis, transferred to polyvinylidene difluoride membranes (Merck Millipore), blocked with Blocking One (Nacalai Tesque, Kyoto, Japan), and washed with TBST (TBS with 0.1% Tween20). The blots were incubated with the primary antibodies shown in Supplementary Table S1, and reaction products were visualized using a horseradish peroxidase-conjugated secondary antibody (Jackson Immuno Research, West Grove, PA, United States) and enhanced chemiluminescence (Thermo Fisher Scientific). Each protein was detected by LAS 3000 (Fujifilm, Tokyo, Japan). Band densities were measured with Multi Gauge software Ver3.0 (Fujifilm).

### Lipid analysis

Intramyocellular lipid extraction was performed using a previously reported method (Kakehi et al., 2021). Briefly, plantaris muscle tissue was homogenized with 500 μL of saline solution and resuspended in 1,875 μL of ice-cold chloroform/methanol (1:2), then incubated for 15 min on ice and briefly vortexed. After centrifugation, separation of aqueous and organic phases was performed by adding 625 μL of ice-cold saline solution and 625 μL of ice-cold chloroform. The resultant organic phase was dried under N_2_ and re-dissolved in chloroform/methanol (1:2). Triacylglycerol (TG), DG, and ceramides were separated from an aliquot of the total lipid extract by thin-layer chromatography, and then separately eluted DG and ceramides were subsequently analyzed by high-performance liquid chromatography (HPLC). An Agilent HPLC system coupled with an LCQ Deca Ion Trap Mass Spectrometer (Thermo Finnigan, CA, United States) was used. Levels of individual lipids were quantified using spiked internal standards, specifically DG Internal Standard Mixture and Ceramide Internal Standard Mixture, which were obtained from Avanti Polar Lipids (Alabaster, AL, United States). Extracted lipids were analyzed using a method involving HPLC, electrospray mass ionization, and multiple reaction monitoring. Lipids of interest were normalized with muscle wet weight to ensure equal and accurate comparison between treatments.

### PTP1B activity assay

PTP1B activity was assayed by a p-nitrophenol phosphate (PNPP) hydrolysis method. Briefly, plantaris muscles were removed and homogenized in a RIPA buffer. The lysates were centrifuged at 15,000 ×*g* for 25 min at 4°C. PTP1B was immunoprecipitated with anti-PTP1B antibody. The immunoprecipitated samples were incubated in a phosphatase reaction buffer (20 mmol/L HEPES, 150 mmol/L NaCl, 5 mmol/L dithiothreitol, 1 mmol/L PNPP, pH 7.4) for 20 min at 37°C. The reactions were stopped with 0.2 mol/L NaOH, and the absorbance was measured at 450 nm. The assay was run in triplicate.

### RNA isolation and real-time quantitative PCR

Mice were euthanized 24 h after immobilization. The plantaris muscles were removed, immediately frozen in liquid nitrogen, and stored at −80°C. First-strand cDNA generation and real-time PCR were performed as previously described (Kakehi et al., 2016). Briefly, each muscle was homogenized with 0.5 mL of TRIzol Reagent using a TissueLyzer (Qiagen, Hilden, Germany). Total RNA from muscle samples was isolated using the RNeasy Lipid Tissue Mini Kit (Qiagen). One microgram of total RNA was reverse transcribed to cDNA with the High-Capacity cDNA Reverse Transcription (RT) Kit (Thermo Fisher Scientific). Following RT, samples were stored at −30°C for PCR reactions. Real-time PCR was performed using the protocols and detection systems of the ABI Prism 7,500 Fast Sequence Detection System (Thermo Fisher Scientific). PCR products were detected using Fast SYBR^®^ Green Master Mix (Thermo Fisher Scientific) and normalized to TBP expression. Primers were designed using Primer-Blast (NCBI, Bethesda, MD, United States) and the following sequences: PTP1B, 5′- CAC​AGC​GTG​AGC​AGC​ATG​AGT​CC (forward) and 5′- AGA​CCG​CAT​CCT​AAG​CTG​GGC​A (reverse); tumor necrosis factor-alpha (TNFα), 5′-TGC​CAC​AAC​CCA​ACC​AGT​CTC​A (forward) and 5′-AGC​AGT​CTC​CAG​CAG​CCC​AAA​G (reverse); TATA-binding protein (TBP), 5′-ATC​CCA​GGC​CGA​CTA​AAT​CA (forward) and 5′-TTT​CAG​AGC​ATT​GGC​CAT​AGA​A (reverse). The −ΔΔCT method was used to calculate the relative expression ratio (2^−ΔΔCT^) based on the change in threshold values. Normalization of the target genes with an endogenous standard was performed based on the expression of the reference gene (TBP).

### Statistics

All data are expressed as means ± SD. Differences among groups were analyzed by one-way analysis of variance (ANOVA) with Tukey multiple comparisons. A *t*-test was used to analyze differences in body weight and muscle weight. A *p*-value of less than 0.05 was considered to denote a statistically significant difference.

## Results

### Effects of HCI on insulin-induced glucose uptake and intramyocellular lipids in plantaris muscle

We divided mice into NFD and HFD groups. After administering each diet for 2 weeks, we performed the HCI procedure for 24 h. Then, we isolated the plantaris muscle from both legs, i.e., one leg with HCI and the other without. As shown in Table 1, body weight and the wet weight of the bilateral plantaris muscles were higher in the HFD group than in the NFD group (body weight: *p* = 0.015, Plantaris wet weight control: *p* = 0.021, HCI:*p* = 0.03). For each diet, there was no difference in muscle weight before or after 24-h HCI (Table 1).

**TABLE 1:** Body weight and muscle wet weight after each intervention.

		NFD	HFD
Weight (g)		24.4 ± 1.2	27.5 ± 2.2*
Plantaris wet weight (mg)	control	14.9 ± 1.1	16.5 ± 0.8*
	HCI	14.7 ± 2.0	16.4 ± 1.9*

n = 5 in each group, *p < 0.05 vs NFD.

**FIGURE 1:**
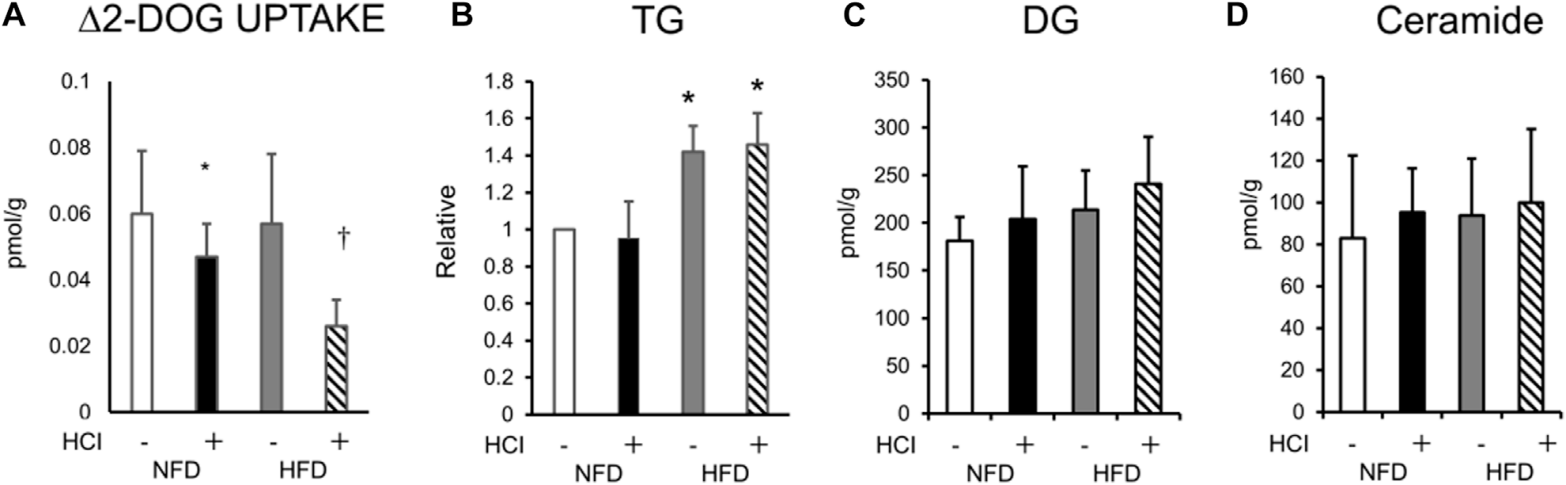
Changes in *ex vivo* insulin-induced 2-DOG uptake and lipid composition after HCI and HFD in plantaris muscle. **(A)** Changes in insulin-induced 2-DOG uptake (Δ2-DOG). ∗NFD with HCI vs NFD without HCI (*p* = 0.041) and HFD without HCI (*p* = 0.044). †HFD with HCI vs NFD without HCI (*p* = 0.02) and HFD without HCI (*p* = 0.023) and NFD with HCI (*p* = 0.046). **(B)**Total TG ∗HFD without HCI vs NFD without HCI (*p* = 0.022) and NFD with HCI (*p* = 0.027), HFD with HCI vs NFD without HCI (*p* = 0.029) and NFD with HCI (*p* = 0.03)., **(C)**DG, and **(D)**ceramide content in plantaris muscle after each treatment. Data are shown as the means ± SD of eight mice per group. **(A–D)** Data are expressed relative to control groups (NFD without HCI: white bar; NFD with HCI: black bar; HFD without HCI: Gy bar; HFD with HCI: striped bar). *p* values were determined by ANOVA followed by Tukey multiple comparison tests.

Next, we measured *ex vivo* insulin-induced muscle glucose uptake in each group using the plantaris muscles isolated from both legs (with and without HCI). While insulin-induced muscle glucose uptake in legs without HCI was comparable between the HFD and NFD groups, that in legs with HCI was significantly lower than in legs without HCI after NFD, and further reduction of insulin-stimulated muscle glucose uptake was observed in legs with HCI after HFD (Figure 1A). As we previously found that HCI resulted in the accumulation of intramyocellular DG in soleus muscle, we investigated intramyocellular TG, DG, and ceramide content in plantaris muscle. Although TG content was higher in the HFD group than in the NFD group, it was unaffected by HCI (Figure 1B). Total intramyocellular DG and ceramide content were comparable in each group (Figures 1C,D). We then investigated specific DG and ceramide components. As shown in Supplementary Figure S1, the amounts of two species of DG (18:1–18:2, 18:1–18:1) were higher in the HCI leg of the HFD group than in both legs of the NFD group or the control leg of the HFD group; however, the amounts of these species in the HCL leg of the NFD group were not different from those in any other legs. As shown in Supplementary Figure S2, the amounts of all species of intramyocellular ceramide were comparable between the four groups. These results suggest that unlike previous results in soleus muscle (Kakehi et al., 2021), there were no changes in the amounts of the intracellular lipids DG and ceramide, which are deeply involved in the development of insulin resistance (Hage Hassan et al., 2014; Metcalfe et al., 2018; Lee et al., 2022).

### Effect of HCI and HFD on the insulin signaling pathway in plantaris muscle

**FIGURE 2:**
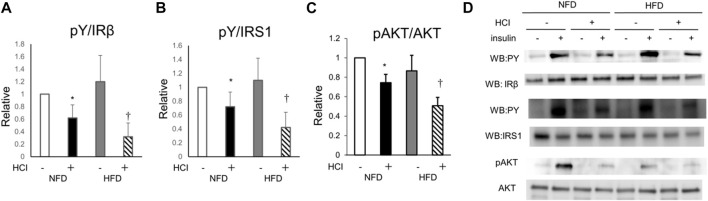
Changes in insulin signal transduction by HCI and HFD in plantaris muscle. **(A)** Quantification of insulin-induced tyrosine phosphorylation of IR relative to IR protein. ∗NFD with HCI vs NFD without HCI (*p* = 0.028) and HFD without HCI (*p* = 0.045). †HFD with HCI vs NFD without HCI (*p* = 0.02) and HFD without HCI (*p* = 0.038) and NFD with HCI (*p* = 0.043). **(B)** Quantification of insulin-induced tyrosine phosphorylation of IRS1 relative to IRS1 protein. ∗NFD with HCI vs NFD without HCI (*p* = 0.03) and HFD without HCI (*p* = 0.042). †HFD with HCI vs NFD without HCI (*p* = 0.013) and HFD without HCI (*p* = 0.048) and NFD with HCI (*p* = 0.038). **(C)** Quantification of insulin-induced phosphorylation of Akt at Ser478 relative to Akt protein. ∗NFD with HCI vs NFD without HCI (*p* = 0.019) and HFD without HCI (*p* = 0.031). †HFD with HCI vs NFD without HCI (*p* = 0.008) and HFD without HCI (*p* = 0.012) and NFD with HCI (*p* = 0.03). **(D)** Representative immunoblots of insulin-induced insulin signaling proteins. Data are shown as the means ± SD of 8–10 mice per group. **(A–C)** Data are expressed relative to control groups (NFD without HCI: white bar; NFD with HCI: black bar; HFD without HCI: Gy bar; HFD with HCI: striped bar). *p* values were determined ANOVA followed by Tukey multiple comparison tests.

**FIGURE 3:**
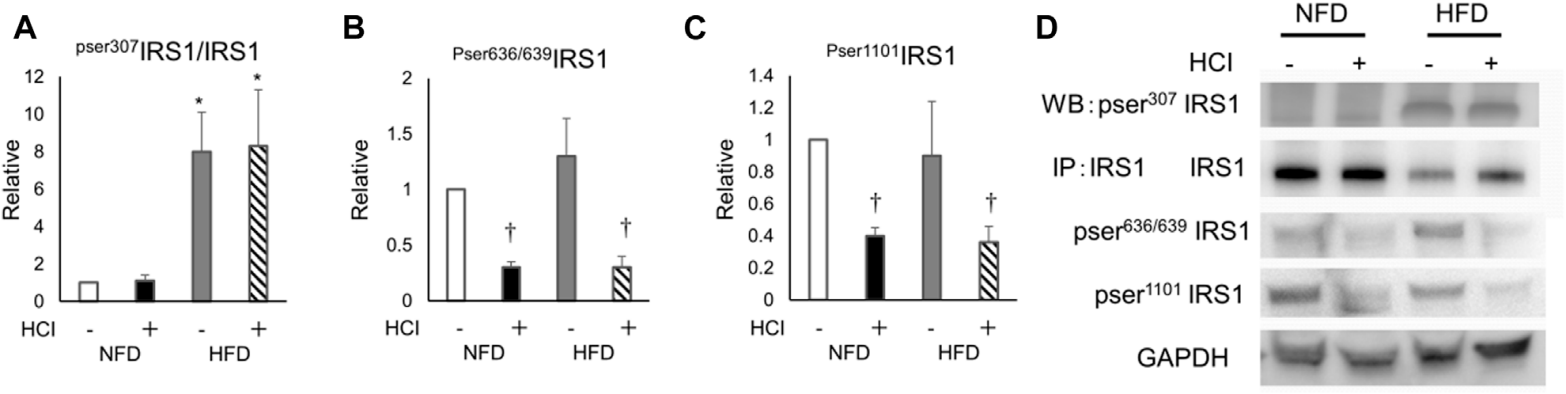
The effects of HCI and HFD on IRS1 phosphorylation level in plantaris muscle. **(A)** Quantification of Ser307 phosphorylation in IRS1 ∗HFD without HCI vs NFD without HCI (*p* = 0.01) and NFD with HCI (*p* = 0.018), HFD with HCI vs NFD without HCI (*p* = 0.02) and NFD with HCI (*p* = 0.022) **(B)** Quantification of Ser636/639 phosphorylation in IRS1 ∗NFD with HCI vs NFD without HCI (*p* = 0.022) and HFD without HCI (*p* = 0.024), HFD with HCI vs NFD without HCI (*p* = 0.025) and HFD without HCI (*p* = 0.03) **(C)** Quantification of Ser1101 phosphorylation in IRS1∗NFD with HCI vs NFD without HCI (*p* = 0.023) and HFD without HCI (*p* = 0.04), HFD with HCI vs NFD without HCI (*p* = 0.025) and HFD without HCI (*p* = 0.03) **(D)** Representative immunoblots of IRS1 proteins. Data are shown as the means ± SD of six mice per group. **(A–C)** Data are expressed relative to control groups (NFD without HCI: white bar; NFD with HCI: black bar; HFD without HCI: gray bar; HFD with HCI: striped bar). *p* values were determined by ANOVA followed by Tukey multiple comparison tests.

To clarify how HCI induces insulin resistance by mechanisms other than intracellular lipids, we analyzed AKT phosphorylation, which occurs downstream of insulin signaling. AKT phosphorylation was reduced by HCI after NFD and further downregulated by HCI after HFD. These changes paralleled insulin-induced muscle glucose uptake levels in each group (Figures 2C,D). Furthermore, the suppressed insulin-stimulated AKT phosphorylation was closely associated with impaired insulin-stimulated phosphorylation levels of IRS1 and its upstream IR (Figures 2A,B,D). Serine phosphorylation of IRS1 at 307, 636/639, and 1101 are known to be associated with impairment of insulin signaling at IRS1 (Summers and Nelson, 2005; Morino et al., 2006); Ser307 in IRS1 is regulated by JNK (Lee et al., 2003) or PKCθ (Yu et al., 2002), and both Ser636/639 (Kang et al., 2011) and Ser1101 (Tremblay et al., 2007) are regulated by S6K. The phosphorylation level of Ser307 was increased in the HFD group (Figures 3A,D), but its phosphorylation level was unaltered by HCI. By contrast, the phosphorylation levels of Ser636/639 and Ser1101 were decreased rather than increased by HCI in both groups (Figures 3B–D). Thus, IRS1 serine phosphorylation levels were not associated with impaired tyrosine phosphorylation of IRS1 by HCI in this study, suggesting that plantaris muscle insulin resistance caused by HCI was likely due to impaired tyrosine phosphorylation at the IR or IRS1 level.

### PTP1B in HCI and HFD contributes to decreased IR phosphorylation levels in plantaris muscle

**FIGURE 4:**
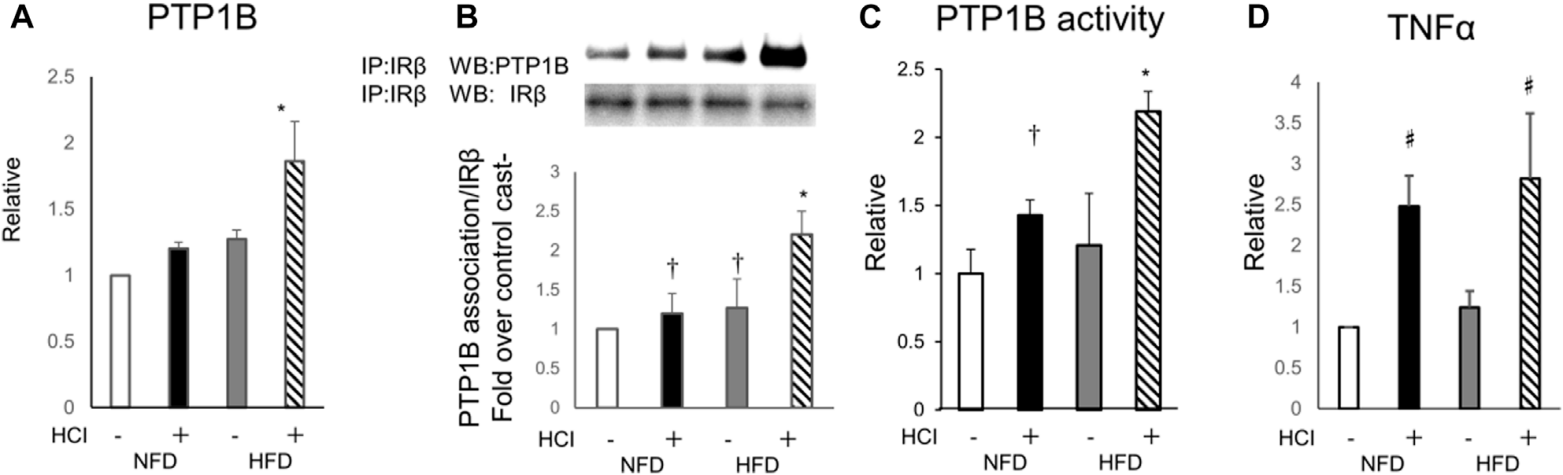
Involvement of PTP1B in the effects of HFD and HCI in plantaris muscle. **(A)** Quantification of PTP1B mRNA levels relative to TBP mRNA. ∗HFD with HCI vs NFD without HCI (*p* = 0.011) and NFD with HCI (*p* = 0.024) and HFD without HCI (*p* = 0.026) **(B)** Representative immunoblots and quantitation of PTP1B proteins immunoprecipitated with IRβ. Quantification of PTP1B relative to IRβ protein. ∗HFD with HCI vs NFD without HCI (*p* = 0.013) and NFD with HCI (*p* = 0.024) and HFD without HCI (*p* = 0.031), †NFD with HCI vs NFD without HCI (*p* = 0.041) and HFD without HCI vs NFD without HCI (*p* = 0.044) **(C)** PTP1B activity. ∗HFD with HCI vs NFD without HCI (*p* = 0.01) and NFD with HCI (*p* = 0.034) and HFD without HCI (*p* = 0.037), †NFD with HCI vs NFD without HCI (*p* = 0.033) **(D)** Quantification of TNFα mRNA levels relative to TBP mRNA. Data are expressed relative to control groups. #NFD with HCI vs NFD without HCI (*p* = 0.008) and HFD without HCI (*p* = 0.01), and HFD with HCI vs NFD without HCI (*p* = 0.012) and HFD without HCI (*p* = 0.026) **(A–D)** Data are shown as the means ± SD of 8–15 mice per group (NFD without HCI: white bar; NFD with HCI: black bar; HFD without HCI: Gy bar, HFD with HCI: striped bar). *p* values were determined by ANOVA followed by Tukey multiple comparison tests.

PTP1B, a negative regulator of the insulin signaling cascade that acts as an IR phosphatase, is upregulated by HFD in various tissues, including skeletal muscle, and is thought to be one of the causes of HFD-induced insulin resistance (Andersen et al., 2001; Zabolotny et al., 2008). Thus, we evaluated the expression of PTP1B and its activity to determine whether tyrosine phosphorylation is inhibited at the IR level which is further upstream. Although 2 weeks of HFD alone did not alter PTP1B expression in the plantaris muscle, HCI after HFD increased PTP1B expression two-fold (Figure 4A). Next, we examined the molecular interaction between IR and PTP1B, which is negatively regulating the level of tyrosine phosphorylation of IR by insulin (Ropelle et al., 2006). Both HCI and HFD promoted the interaction, which was further enhanced by HCI after HFD (Figure 4B). In addition, PTP1B dephosphorylation was increased by HCI and further enhanced by HCI after HFD (Figure 4C). Furthermore, the expression level of TNFα, which activates PTP1B, was increased by approximately two-fold by HCI in both groups (Figure 4D). By contrast, HCI did not alter PTP1B activity or the molecular interaction between IR and PTP1B in soleus muscle (Supplementary Figure S3A,B). These findings suggest that the large increase in PTP1B activity following HCI of the plantaris muscle and administration of HFD might be caused by the synergistic effects of the HFD- and HCI-induced increase in PTP1B expression and the HCI-induced increase in TNFα expression, whereas the same intervention did not alter PTP1B activity in soleus muscle.

### Effect of a PTP1B inhibitor on the suppression of insulin-induced AKT and IR phosphorylation by HCI and HFD

**FIGURE 5:**
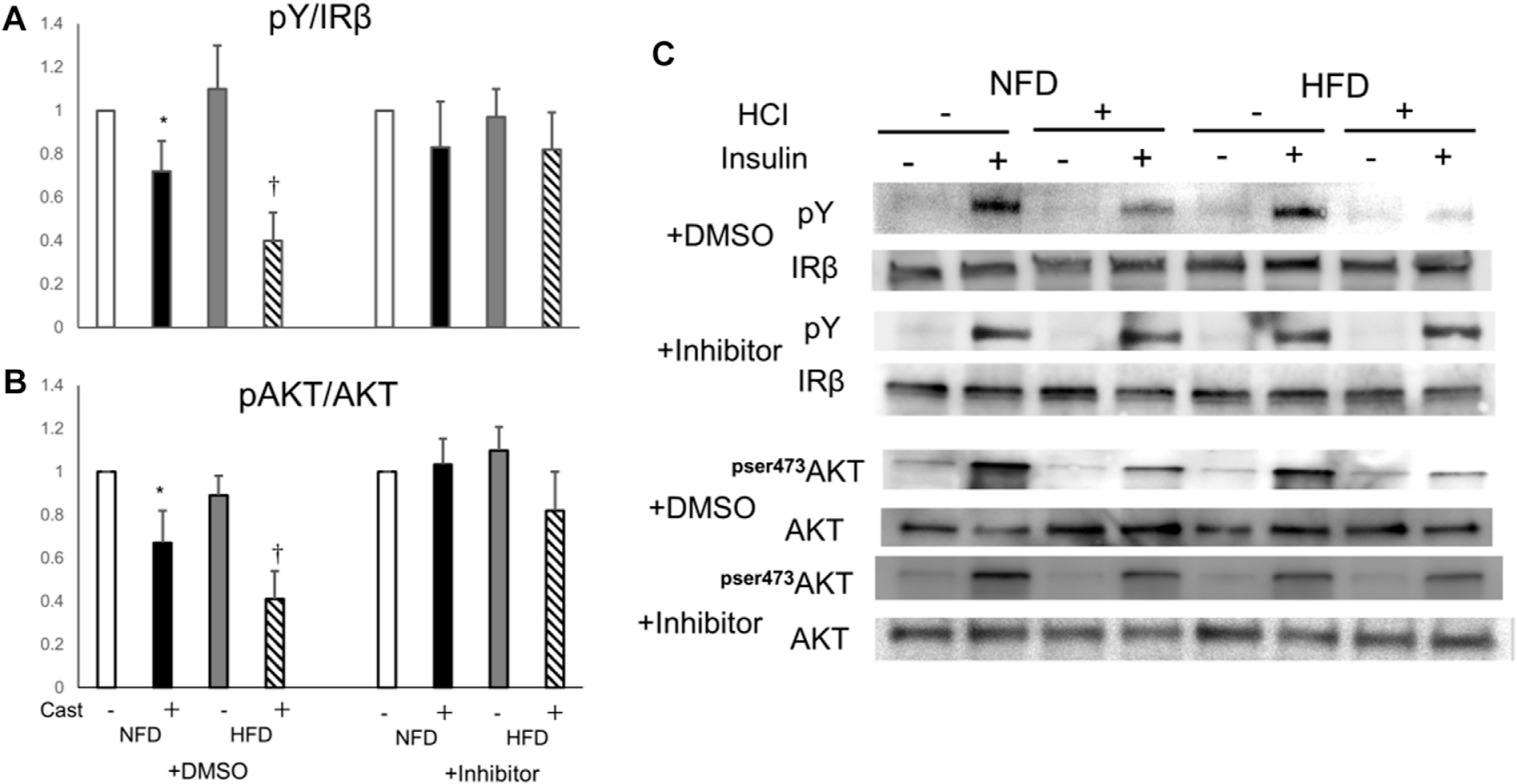
Effects of PTP1B inhibition on insulin signaling suppression caused by HFD and HCI in plantaris muscle. PTP1B inhibition was performed by preincubating 10 μM PTP1B cell-permeable inhibitor with dissected plantaris muscle for 30 min at 37°C. **(A)** Quantification of insulin-induced tyrosine phosphorylation of IR relative to IR protein. ∗NFD with HCI vs NFD without HCI (*p* = 0.036) and HFD without HCI (*p* = 0.041). †HFD with HCI vs NFD without HCI (*p* = 0.014) and HFD without HCI (*p* = 0.037) and NFD with HCI (*p* = 0.043). **(B)** Quantification of insulin-induced phosphorylation of Akt at Ser478 relative to Akt protein. ∗NFD with HCI vs NFD without HCI (*p* = 0.033) and HFD without HCI (*p* = 0.04). †HFD with HCI vs NFD without HCI (*p* = 0.021) and HFD without HCI (*p* = 0.031) and NFD with HCI (*p* = 0.049). **(C)** Representative immunoblots of insulin-induced insulin signaling proteins. **(A, B)** Data are expressed relative to control groups. Data are shown as the means ± SD of 8–15 mice per group (NFD without HCI: white bar; NFD with HCI: black bar; HFD without HCI: Gy bar, HFD with HCI: striped bar). *p* values were determined by ANOVA followed by Tukey multiple comparison tests.

Finally, to confirm the involvement of PTP1B in the mechanism of HFD- and HCI-induced insulin resistance, we evaluated insulin signaling in plantaris muscle treated with a PTP1B inhibitor after HFD and HCI. The addition of the PTP1B inhibitor almost completely counteracted the reduction in insulin-stimulated IR and AKT phosphorylation after HCI following NFD or HFD, suggesting that PTP1B activation plays a causal role in HFD- and HCI-induced insulin resistance in plantaris muscle (Figures 5A–C).

## Discussion

This study showed that 24-h HCI caused insulin resistance in the fast-twitch–predominant plantaris muscle and that HFD augmented these effects. However, in contrast to the results reported for the slow-twitch–predominant soleus muscle (Kakehi et al., 2021), accumulation of intramyocellular lipids such as DG and ceramide, which may induce insulin resistance, was rarely observed in plantaris muscle. Instead, the HFD- and HCI-induced insulin resistance in plantaris muscle is likely caused by the activation of PTP1B.

Unlike previous results in soleus muscle, our study demonstrated that intramyocellular TG accumulation in plantaris muscle was increased by HFD, while total DG and ceramide accumulation were not altered by the combination of HFD and HCI. It has been suggested that DG and ceramide are the major molecular species of intramyocellular lipids that contribute to the suppression of insulin signaling (Hage Hassan et al., 2014; Metcalfe et al., 2018; Lee et al., 2022). Our previous study showed that in soleus muscle, HCI suppressed insulin signaling with a increase intramyocellular DG via activation of lipin1 (Kakehi et al., 2021), but these changes were not observed in plantaris muscle. Since plantaris and soleus muscles are mainly composed of fast-twitch and slow-twitch fibers, respectively, the DG-mediated insulin resistance caused by physical inactivity is more likely to occur in slow-twitch fibers than in fast-twitch fibers.

The present study demonstrated that plantaris muscle insulin resistance caused by HFD and HCI was mediated by the activation of PTP1B, which then inhibited key insulin-signaling molecules. Previous studies have shown that diet-induced obesity increases the expression and activation of PTP1B, which inhibits tyrosine phosphorylation of IR and IRS1, and these changes were mostly reversed by exercise (Ropelle et al., 2006). In addition, muscle-specific PTP1B deletion mice were protected against HFD-induced insulin resistance in muscle via increased phosphorylation of IR and its downstream signaling components (Delibegovic et al., 2007). In the present study, inhibition of insulin signaling by HFD and physical inactivity was associated with reduced signaling at the IR level and by PTP1B activation; these changes were completely reversed by a PTP1B inhibitor. In addition, PTP1B activation was not observed in soleus muscle after HCI. Thus, inhibition of insulin signaling at the IR level could be the main mechanism of insulin resistance caused by physical inactivity in the fast-twitch–predominant plantaris muscle, but not in the slow-twitch–predominant soleus muscle.

HCI-induced PTP1B activation might be partly caused by TNFα. The proinflammatory cytokines TNFα (Xu et al., 2008), IL1, and IL6 (Myers et al., 2001; Xu et al., 2008) are locally increased in obesity, and cause PTP1B activation and insulin resistance. Consistent with this study, hindlimb suspension is known to increase TNFα in unloaded skeletal muscle (Hirose et al., 2008). The notably augmented PTP1B activity observed in the HCI with HFD group could be attributed to an increase in PTP1B protein levels. Although we did not directly measure PTP1B protein levels in this study, such an increase, when combined with PTP1B activation via HCI and HFD, could heighten the overall PTP1B activity. Previous studies have also suggested that PTP1B activation could be influenced by JNK, functioning downstream of TNFα (Henstridge et al., 2012; Yang et al., 2012). On a different note, TNFα signaling has been reported to influence the choice of fiber type-specific signaling pathways, leading to an upregulation of JNK-related signals exclusively in fast-twitch muscle fibers (Phillips and Leeuwenburgh, 2005). Therefore, TNFα, which is found to be upregulated in HCI, may trigger the activation of PTP1B via JNK specifically in fast-twitch muscles. This could subsequently result in the inhibition of insulin signaling at the insulin receptor level, offering a potential mechanistic explanation for the observed phenomena.

We acknowledge a limitation of our study related to terminology. The term “physical inactivity” used in our research may be more accurately described as “muscle disuse,” given our model of hindlimb immobilization. We appreciate this nuance and will consider it in future research, focusing on the specific impacts of muscle disuse and broader physical inactivity on muscle physiology and insulin signaling.

In conclusion, our study demonstrates that 24-h HCI causes insulin resistance in the fast-twitch–predominant plantaris muscle and that HFD augments these effects. The mechanism is mediated by the inhibition of IR phosphorylation by PTP1B. This mechanism is novel and differs from that by which HCI causes insulin resistance in the slow-twitch–predominant soleus muscle.

## Data Availability

The original contributions presented in the study are included in the article/Supplementary Material, further inquiries can be directed to the corresponding author.

## Supplementary material

SUPPLEMENTARY FIGURE S1 Intramyocellular DG composition in plantaris muscle after HCI and HFD. DG content in plantaris muscle after each treatment. Data are shown as the means ± SD of eight mice per group. Data are expressed relative to control groups (NFD without HCI: white bar; NFD with HCI: black bar; HFD without HCI: gray bar; HFD with HCI: striped bar). *P < 0.05 vs. all groups. P values were determined by ANOVA followed by Tukey multiple comparison tests.

SUPPLEMENTARY FIGURE S2 Intramyocellular ceramide composition in plantaris muscle after HCI and HFD. Ceramide content in plantaris muscle after each treatment. Data are shown as the means ± SD of eight mice per group. Data are expressed relative to control groups (NFD without HCI: white bar; NFD with HCI: black bar; HFD without HCI: gray bar; HFD with HCI: striped bar).

SUPPLEMENTARY FIGURE S3 Changes in PTP1B activity caused by HCI and HFD in soleus muscle. **(A)** Representative immunoblots and quantitation of PTP1B proteins immunoprecipitated with IRβ. Quantification of PTP1B relative to IRβ protein. **(B)** PTP1B activity. Data are expressed relative to control groups. **(A, B)** Data are shown as the means ± SD of 8–15 mice per group (NFD without HCI: white bar; NFD with HCI: black bar; HFD without HCI: gray bar, HFD with HCI: striped bar).
