# Supplementary material for: Physical inactivity induces insulin resistance in plantaris muscle through protein tyrosine phosphatase 1B activation in mice
Source: Front Physiol. 2023 Jun 14;14:1198390. doi: 10.3389/fphys.2023.1198390 (PMC10300557; doi:10.3389/fphys.2023.1198390)
Supplement: Supplementary file 1 [file Table1.DOCX]

**Supplemental Table 1. Primary Antibodies**

| Product Name | Catalog Number | Company |
| --- | --- | --- |
| anti–insulin receptor substrate 1(IRS1) | 06-248 | Merck Millipore |
| anti-AKT | 9272 | Cell Signaling Technology |
| anti–phospho-AKT (ser473) | 9271 | Cell Signaling Technology |
| anti–phospho-IRS1 (ser307) | 05-1087 | Merck Millipore |
| anti–phospho-IRS1 (Ser636/639) | 2388 | Cell Signaling Technology |
| Anti–-phospho-IRS1 (Ser1101) | 2385 | Cell Signaling Technology |
| anti–protein tyrosine phosphatase 1B | ab252928 | Abcam |
| anti–insulin receptor β | 3025 | Cell Signaling Technology |
