# Supplementary material for: Physical inactivity induces insulin resistance in plantaris muscle through protein tyrosine phosphatase 1B activation in mice
Source: Front Physiol. 2023 Jun 14;14:1198390. doi: 10.3389/fphys.2023.1198390 (PMC10300557; doi:10.3389/fphys.2023.1198390)
Supplement: Supplementary file 2 [file Presentation1.PPTX]

## Slide 1
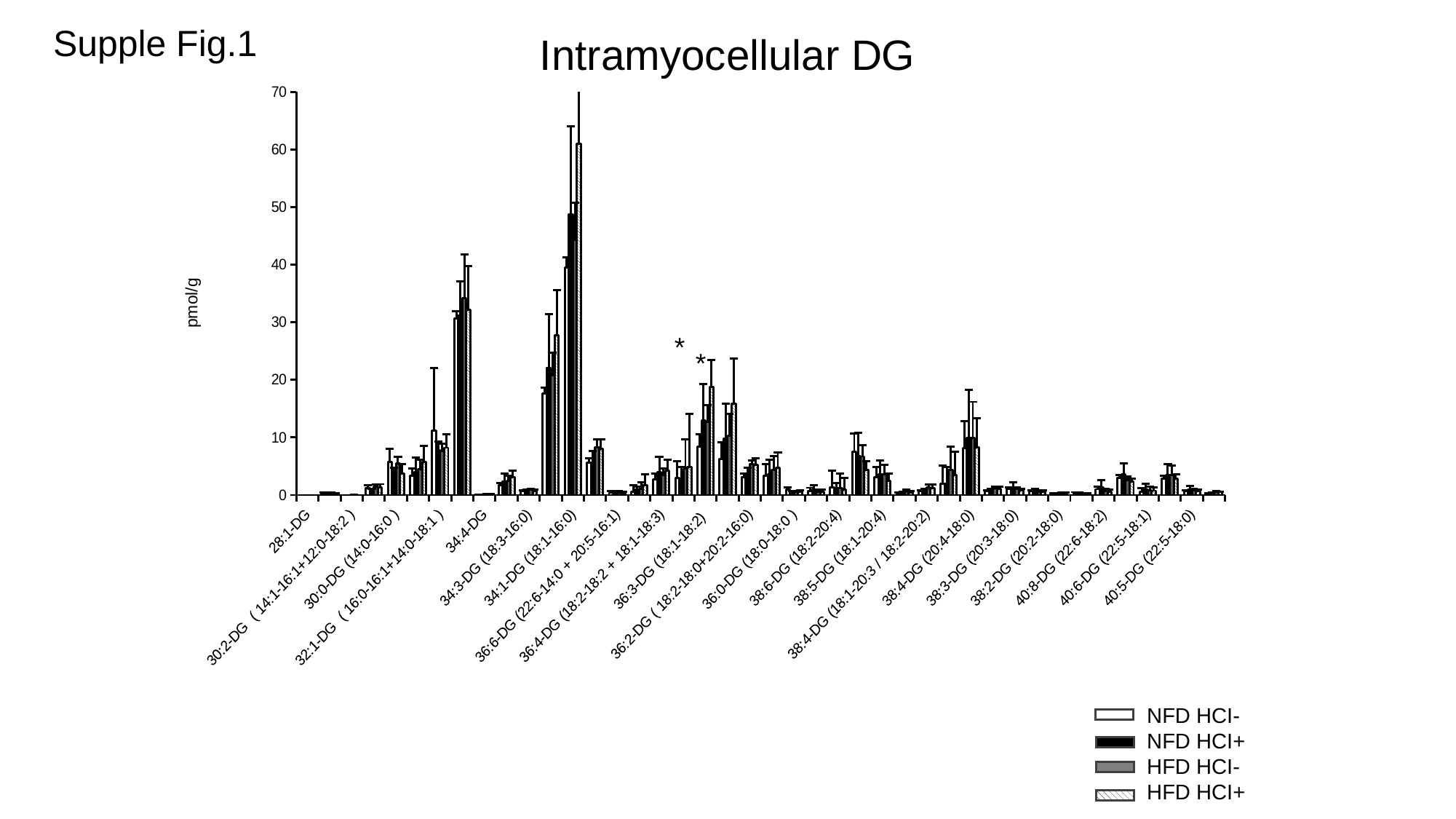

Supple Fig.1
Intramyocellular DG
### Chart
| Category | | | | |
|---|---|---|---|---|
| 28:1-DG | 0.0 | 0.0 | 0.0 | 0.0 |
| 28:0-DG ( 14:0-14:0 ) | 0.23484515753142476 | 0.20699297989642662 | 0.25857223559044584 | 0.15433665769807983 |
| 30:2-DG ( 14:1-16:1+12:0-18:2 ) | 0.0 | 0.0 | 0.02983649600190953 | 0.0 |
| 30:1-DG ( 14:0-16:1+12:0-18:1+16:0-14:1 ) | 1.1545827762470005 | 0.7916782307248627 | 1.389756224800432 | 1.2879092849317895 |
| 30:0-DG (14:0-16:0 ) | 5.748805251964695 | 3.5337821738296644 | 5.528880624743566 | 3.7744164368611255 |
| 32:2-DG ( 16:1-16:1+14:0-18:2+16:0-16:2 ) | 3.323434633294812 | 3.9922767687850524 | 4.534722917105592 | 5.759782300475724 |
| 32:1-DG ( 16:0-16:1+14:0-18:1 ) | 11.163753788593416 | 7.83642813492481 | 7.599583058366126 | 8.212057739924788 |
| 32:0-DG ( 16:0-16:0 ) | 30.617825983664453 | 31.214469512361003 | 34.253053358618324 | 32.17849706730043 |
| 34:4-DG | 0.012941106871358004 | 0.030663780663780664 | 0.04833099979275507 | 0.04931315727729446 |
| 34:3-DG (18:2-16:1) | 1.6771592807037174 | 2.396642954599116 | 2.4068131635263934 | 3.146708756904553 |
| 34:3-DG (18:3-16:0) | 0.6715486185002204 | 0.6468965324276404 | 0.8892193676799812 | 0.6935195962258502 |
| 34:2-DG ( 18:1-16:1+18:2-16:0 ) | 17.618397380773697 | 22.131481417429967 | 20.791949555074233 | 27.76894938535102 |
| 34:1-DG (18:1-16:0) | 39.56022869212117 | 48.75238484602929 | 44.351460911961354 | 60.959780931839504 |
| 34:0-DG (18:0-16:0) | 5.60162284857311 | 5.541620344442172 | 8.254591077930622 | 8.077262970251056 |
| 36:6-DG (22:6-14:0 + 20:5-16:1) | 0.3776693180543111 | 0.3624033449505116 | 0.4358790734464212 | 0.2864982447648682 |
| 36:5-DG (18:3-18:2) | 0.61393231758476 | 0.9801536211024235 | 1.0249337842453976 | 1.6461683037890524 |
| 36:4-DG (18:2-18:2 + 18:1-18:3) | 2.728323610591745 | 3.941500889958518 | 3.459105647575832 | 4.195946719967498 |
| 36:4-DG ( 20:4-16:0 ) | 2.945787077050252 | 2.5644490416744063 | 4.548285068448671 | 4.8220493036712915 |
| 36:3-DG (18:1-18:2) | 8.376526027614535 | 12.969953571529134 | 12.710889541568502 | 18.726577332182185 |
| 36:2-DG ( 18:1-18:1) | 6.248629380299744 | 9.820129901058323 | 10.341083466947886 | 15.869586473424006 |
| 36:2-DG ( 18:2-18:0+20:2-16:0) | 3.109998252136299 | 3.417834443066322 | 5.354973819160561 | 5.193867043766588 |
| 36:1-DG (18:1-18:0 + 20:1-16:0) | 3.3720280859370595 | 3.627207426042056 | 4.392761388847313 | 4.793234734689586 |
| 36:0-DG (18:0-18:0 ) | 0.970481564182033 | 0.378803149520025 | 0.6033749665305375 | 0.6179181306173595 |
| 38:7-DG (22:6-16:1) | 0.7078247829821115 | 0.911348797211434 | 0.5255695328175832 | 0.5899497056421696 |
| 38:6-DG (18:2-20:4) | 1.281973956144288 | 0.7514113098199291 | 1.220082419698375 | 0.9667119190289215 |
| 38:6-DG (22:6-16:0) | 7.54902505502543 | 6.820492398455053 | 6.695509885932556 | 4.3244239987112385 |
| 38:5-DG (18:1-20:4) | 3.1165035750052494 | 3.5824005723706427 | 3.5404599043145586 | 2.439162498431885 |
| 38:5-DG (22:5-16:0) | 0.3342534207155271 | 0.3302136937588571 | 0.762546997600573 | 0.4217582789236832 |
| 38:4-DG (18:1-20:3 / 18:2-20:2) | 0.7131355165242802 | 0.630065837812711 | 1.2997451723878604 | 1.2089566495862556 |
| 38:4-DG (22:4-16:0) | 1.9003270515964887 | 1.9840203079724685 | 4.331440227368674 | 3.468689426034446 |
| 38:4-DG (20:4-18:0) | 8.168888052074273 | 9.958971018863283 | 9.926630175063478 | 8.278587529561758 |
| 38:3-DG (18:2-20:1 / 18:1-20:2) | 0.6945361630240634 | 0.8400125596649356 | 1.1797498772925261 | 1.0543323703133773 |
| 38:3-DG (20:3-18:0) | 1.0314792895976308 | 1.2958454649749291 | 1.0149786541543215 | 0.8209944419604184 |
| 38:2-DG (18:1-20:1) | 0.6283790185634024 | 0.602905196885727 | 0.5947561611412236 | 0.609794371730032 |
| 38:2-DG (20:2-18:0) | 0.2096783505546521 | 0.1803671738807974 | 0.3576719237492485 | 0.2832815983821319 |
| 38:1-DG (20:1-18:0/18:1-20:0) | 0.30621555596211264 | 0.26417522200620797 | 0.2649332962793551 | 0.2324807963455969 |
| 40:8-DG (22:6-18:2) | 1.011102991793946 | 1.3515751619514211 | 0.6349388753558561 | 0.5790539117113163 |
| 40:7-DG (22:6-18:1) | 2.932214428493725 | 3.603246588103944 | 2.5646049016358976 | 2.363578770284797 |
| 40:6-DG (22:5-18:1) | 0.6016970918909949 | 0.9430165824993711 | 0.8389117993917907 | 0.754893266155302 |
| 40:6-DG (22:6-18:0) | 2.83879207091521 | 3.5094083819558564 | 3.4638027352777234 | 2.899658106203723 |
| 40:5-DG (22:5-18:0) | 0.7097605274864538 | 0.9331414563947191 | 0.8308518598270106 | 0.7501099393134415 |
| 40:4-DG (22:4-18:0) | 0.19878631450937018 | 0.21637192345611259 | 0.6499356636398622 | 0.5108339209433699 |pmol/g
*
*
NFD HCI-
NFD HCI+
HFD HCI-
HFD HCI+

## Slide 2
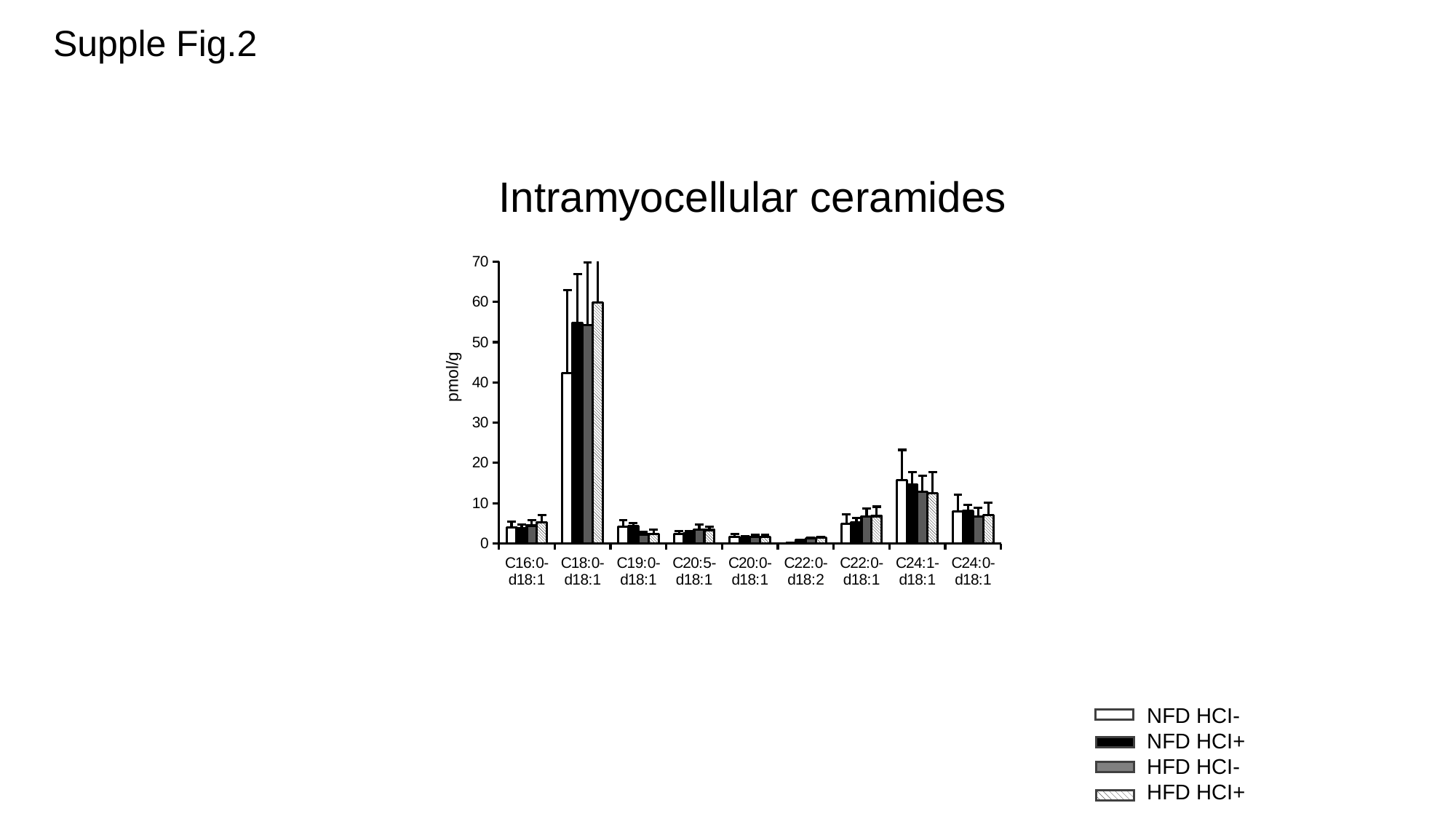

Supple Fig.2
Intramyocellular ceramides
### Chart
| Category | | | | |
|---|---|---|---|---|
| C16:0-d18:1 | 3.9181249329393304 | 3.721183376739286 | 4.432990434300782 | 5.162850328920675 |
| C18:0-d18:1 | 42.31141997597638 | 54.73376142423869 | 54.310999572029566 | 59.771186826097406 |
| C19:0-d18:1 | 4.095924948666117 | 4.315463355821326 | 2.263153743772417 | 2.4190621696835777 |
| C20:5-d18:1 | 2.3332739589405045 | 2.471863823572556 | 3.4841024225508113 | 3.339493319101223 |
| C20:0-d18:1 | 1.588012462769557 | 1.4164178643336502 | 1.6218484423175972 | 1.6753187727318244 |
| C22:0-d18:2 | 0.0 | 0.822071751348067 | 1.2585555604257133 | 1.374994504889717 |
| C22:0-d18:1 | 4.909545167318928 | 5.1583258236799345 | 6.6864765514191955 | 6.782117253232451 |
| C24:1-d18:1 | 15.68128118012493 | 14.556660121200292 | 12.820674512578318 | 12.429559580133391 |
| C24:0-d18:1 | 7.956368335225854 | 8.047662015391314 | 6.7681584942639725 | 6.982822113305314 |pmol/g
NFD HCI-
NFD HCI+
HFD HCI-
HFD HCI+

## Slide 3
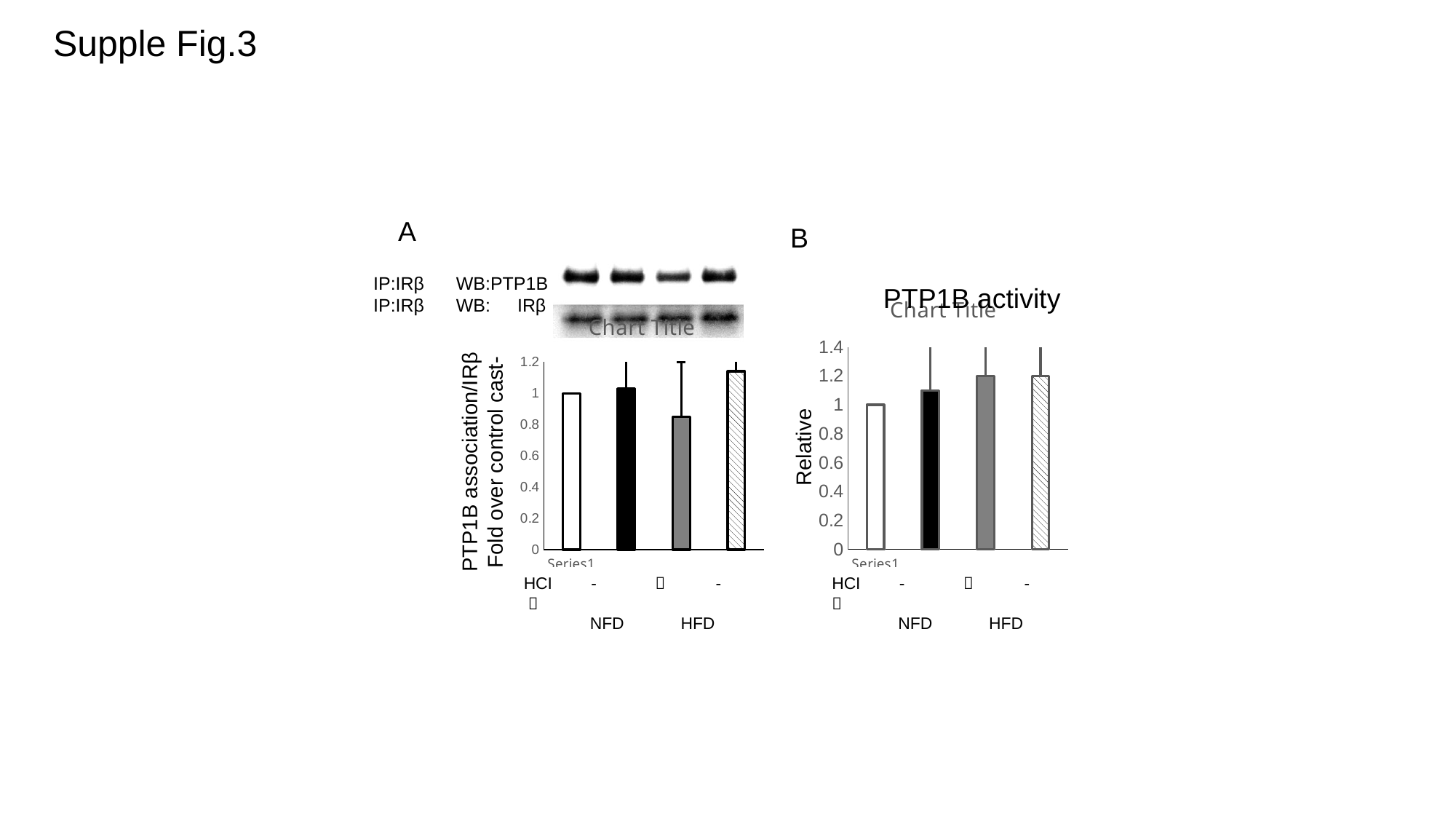

Supple Fig.3
A
B
IP:IRβ　 WB:PTP1B
IP:IRβ　 WB:　IRβ
PTP1B activity
### Chart:
| Category | |
|---|---|
| | 1.0 |
| | 1.1 |
| | 1.2 |
| | 1.2 |
### Chart:
| Category | |
|---|---|
| | 1.0 |
| | 1.03 |
| | 0.85 |
| | 1.14 |Relative
PTP1B association/IRβ
Fold over control cast-
HCI 　 -　　　 ＋　　 -　　 ＋
 NFD HFD
HCI 　 -　　　 ＋　　 -　　 ＋
 NFD HFD
